# A 10-year study reveals clinical and laboratory evidence for the ‘semi-invasive' properties of chronic pulmonary aspergillosis

**DOI:** 10.1038/emi.2016.31

**Published:** 2016-04-20

**Authors:** Jasper Fuk-Woo Chan, Susanna Kar-Pui Lau, Sally Cheuk-Ying Wong, Kelvin Kai-Wang To, Simon Yung-Chun So, Sally Sau-Man Leung, Siu-Mang Chan, Chiu-Mei Pang, Chenlu Xiao, Ivan Fan-Ngai Hung, Vincent Chi-Chung Cheng, Kwok-Yung Yuen, Patrick Chiu-Yat Woo

**Affiliations:** 1 State Key Laboratory of Emerging Infectious Diseases, The University of Hong Kong, Hong Kong, China; 2 Department of Microbiology, The University of Hong Kong, Hong Kong, China; 3 Research Centre of Infection and Immunology, The University of Hong Kong, Hong Kong, China; 4 Carol Yu Centre for Infection, The University of Hong Kong, Hong Kong, China; 5 Department of Medicine, The University of Hong Kong, Hong Kong, China

**Keywords:** aspergilloma, aspergillosis, chronic, invasive, pulmonary

## Abstract

In recent years, infections caused by *Aspergillus* sp. have become an emerging focus of clinical microbiology and infectious disease, as the number of patients infected with *Aspergillus* sp. has increased markedly. Although chronic pulmonary aspergillosis (CPA) is considered a ‘semi-invasive' or ‘intermediate' disease, little data are available for the direct comparison of CPA with invasive pulmonary aspergillosis (IPA) and pulmonary aspergilloma (PA) to quantify invasiveness. In this study, we compared the characteristics of CPA with those of IPA and PA among hospitalized patients over a 10-year period. A total of 29, 51 and 31 cases of CPA, IPA and PA, respectively, were included. An increasing trend in galactomannan antigen seropositivity rate from PA (24.1%) to CPA (35.7%) to IPA (54.9%) and an opposite trend for anti-*Aspergillus* antibody (PA (71.0%) to CPA (45.8%) to IPA (7.1%)) were observed. Eight percent of CPA patients were infected with more than one *Aspergillus* sp. The survival rate of the CPA group also fell between the survival rate of PA and IPA, confirming the intermediate severity of CPA. The survival rate of the CPA group became significantly higher than that of the IPA group from day 180 onwards until 2 years after admission (*P*<0.05). The survival rate of the CPA group remained lower than that of the PA group from day 30 onwards until 2 years after admission. Poor prognostic factors for CPA included older age (*P*=0.019), higher total leukocyte count (*P*=0.011) and higher neutrophil count (*P*=0.012) on admission. This study provided clinical and laboratory evidence for the semi-invasive properties of CPA.

## INTRODUCTION

In recent years, infections caused by *Aspergillus* sp. have become an emerging focus of clinical microbiology and infectious disease, as the number of patients infected with *Aspergillus* sp. has increased dramatically. Pulmonary aspergillosis is the most common form of infection caused by *Aspergillus* sp. and is associated with significant morbidity and mortality in a wide range of susceptible hosts.^1, 2^ Disease evolvement after inhalation of *Aspergillus* spores is mainly determined by the host's systemic and local immune status. Two major non-allergic forms of pulmonary aspergillosis were previously widely recognized: simple pulmonary aspergilloma (PA), which is usually an indolent disease that develops in patients with pre-existing lung cavities, and invasive pulmonary aspergillosis (IPA), which mainly occurs, in severely immunocompromised patients and is associated with a nearly 100% mortality rate in the absence of prompt and effective antifungal treatment.^1^ In the early 1980s, Gefter *et al.*^3^ and Binder *et al.*^4^ independently proposed a new clinical entity, chronic necrotizing pulmonary aspergillosis, as an ‘intermediate' or ‘semi-invasive' form of pulmonary aspergillosis that occurs in patients with chronic lung disease or mild immunosuppression. In 2003, Denning *et al.*^5^ further proposed the additions of chronic cavitary pulmonary aspergillosis and chronic fibrosing pulmonary aspergillosis to, and exclusion of simple PA from, the spectrum of chronic pulmonary aspergillosis (CPA).

Over the past decade, CPA has been increasingly described in case reports and small case series in different ethnic groups.^1^ A major limitation of these published studies on CPA is the lack of objective quantitation of its ‘semi-invasiveness' by direct and systematic comparison of the clinical and laboratory characteristics of CPA with those of IPA and PA in patients from the same population over a unified study period. This has made the accurate assessment of the relative severities of these conditions problematic. Furthermore, the comparative diagnostic performances of non-invasive serological tests, including galactomannan antigen and antibody assays, which are increasingly being used to diagnose the different forms of pulmonary aspergillosis, have not been systemically compared. The positive rates of these non-invasive serological tests were variable in previous studies, possibly due to inter-laboratory variations in methodology and result interpretations. To objectively quantify the severity of CPA, we conducted this 10-year retrospective cohort study to compare the clinical, laboratory and prognostic characteristics of CPA with those of IPA and PA in hospitalized patients from a single tertiary referral center using tests uniformly performed by the same laboratory. This study was the first that systematically compared the characteristics of hospitalized patients with CPA and those with IPA and PA who were managed in the same institution using a constant set of diagnostic criteria and mycological investigations performed by a single laboratory. We objectively assessed the relative severities based on microbiological investigation results and clinical outcome, and we confirmed CPA to be an intermediate form of pulmonary aspergillosis.

## MATERIALS AND METHODS

### Patients and study design

This study was approved by the institutional review board of The University of Hong Kong/Hospital Authority Hong Kong West Cluster in Hong Kong. The electronic case records of hospitalized patients diagnosed with ‘pulmonary aspergillosis' at Queen Mary Hospital, Hong Kong, between 1 July 2003 and 30 June 2013 were retrieved and reviewed. The patients were categorized into three groups according to the previously proposed diagnostic criteria for CPA, IPA and PA (Table 1).^5, 6, 7, 8^ For IPA, only patients with proven, probable or probable IPA without prespecified radiologic findings were included.^6, 7^ Patients whose conditions did not fulfill any of these diagnostic criteria were excluded. The epidemiological, clinical, laboratory, radiological and mycological characteristics were recorded into a predefined database and were compared and analyzed.

### Setting

Queen Mary Hospital, a 1600-bed institution, is the affiliated teaching hospital of The University of Hong Kong, which offers tertiary referral services, including hematopoietic stem cell and solid organ transplantation, cardiopulmonology and intensive care, as well as emergency services for internal medicine, surgery, pediatrics, and obstetrics and gynecology. The patients in this study were not managed in airborne infection isolation rooms unless otherwise specified.

### Mycological investigations

Detection of serum galactomannan antigen and anti-*Aspergillus* antibody was performed using the Platelia *Aspergillus* Ag enzyme immunoassay (Bio-Rad Laboratories, Redmond, WA, USA) and the Meridian Bioscience Fungal Immunodiffusion System (Meridian Bioscience Inc., Cincinnati, OH, USA), respectively, according to the manufacturers' instructions. An index value of ⩾0.5 was considered positive for the serum galactomannan antigen assay. Fungal culture of respiratory tract specimens was performed as previously described.^9^ Briefly, the specimens were processed within 24 h upon their arrival at the clinical microbiology laboratory, and all of the procedures were performed inside a class II biosafety cabinet to avoid possible culture contamination. The specimens were concentrated by centrifugation for 10 min at 2000 rcf, and the pellet was then resuspended in 1 mL of 0.85% NaCl. A heavy inoculum (~0.25–0.5 mL) was then inoculated onto Sabouraud dextrose agar (Sigma-Aldrich, St Louis, MO, USA) supplemented with chloramphenicol (50 μg/mL), and the plate was then incubated at 30 °C until moldy colonies were observed at the primary inoculation sites.

### Statistical analysis

All statistical analyses were performed using PASW Statistics software (version 18.0.0). The Fisher's exact and chi-square tests were used for categorical variables where appropriate, and the Mann–Whitney *U*-test was used for continuous variables. The log-rank test was used to compare survival distributions. To determine whether a parameter was independently associated with death, backward stepwise multivariate regression analysis was used to control for confounding clinical risk conditions. *P-*values <0.05 were considered statistically significant.

## RESULTS

### Patients

**Figure 1:**
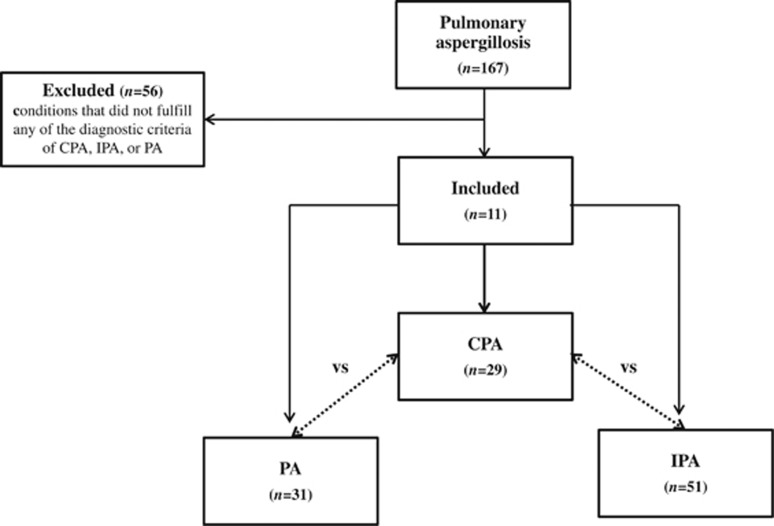
The total number of patients with CPA, IPA and PA, and the reasons for exclusion are shown. chronic pulmonary aspergillosis, CPA; invasive pulmonary aspergillosis, IPA; pulmonary aspergilloma, PA.

A total of 167 patients were diagnosed with ‘*pulmonary aspergillosis*' in the 10-year study period (Figure 1). Fifty-six patients did not fulfill the diagnostic criteria and were excluded, and a total of 29, 51 and 31 patients fulfilled the diagnostic criteria of CPA, IPA and PA, respectively. The demographic characteristics of the patients and their laboratory investigation results are described in Table 2. The median age of the CPA group (64 years) was significantly higher than the IPA group (51 years), but was not significantly different from the PA group. The male to female ratio did not significantly differ between the CPA group and either the IPA group or the PA group. As expected from the case definitions, a higher proportion of the IPA group had systemic immunocompromising conditions, such as neutropenia, hematopoietic stem cell or solid organ transplantation, malignancy, and recent chemotherapy or immunosuppressant exposure, whereas higher proportions of the CPA and PA groups had chronic lung diseases or past pulmonary tuberculosis (Supplementary Table S1).

### Mycological investigations

An increasing trend in the galactomannan antigen seropositivity rate from PA (24.1%) to CPA (35.7%) to IPA (54.9%) and an opposite trend for anti-*Aspergillus* antibody PA (71.0%) to CPA (45.8%) to IPA (7.1%) were observed (Table 2). The respiratory tract specimen cultures (CPA, 86.2% IPA, 63.6% PA, 64.5%) did not differ significantly between the CPA group and either the IPA group or the PA group. *Aspergillus fumigatus* was the most commonly isolated species in all three groups (CPA, 68.0% IPA, 64.3% PA, 50.0%). Nearly one-tenth of the culture-positive CPA patients had more than one species isolated from the respiratory tract specimens, whereas none of the culture-positive IPA and PA patients had more than one *Aspergillus* species isolated from the respiratory tract specimens.

### Radiological findings

As expected from the case definitions, PA patients had a unilobar lesion involving either the upper lobe as viewed in a chest X-ray (CXR) and thoracic computed tomography (CT) scan, whereas over 40% of CPA and IPA patients had lesions in more than one lobe in CXR and thoracic CT scan (Table 3). Most CPA and IPA patients with multilobar lesions had bilateral upper lobe involvement, whereas involvement of the other lobes was less common. Cavitary lesion and consolidation and/or collapse were the most common findings in both CXR and thoracic CT scan in CPA and IPA patients, respectively. Only around one-third of the IPA group had the classical findings of cavitary lesion, halo and/or air-crescent signs. Fibrosis was also more commonly found in the CPA group in both CXR and thoracic CT scan. CXR was much less sensitive than thoracic CT scan in detecting cavitary lesions in both groups because of coexisting radiological abnormalities, including consolidation, collapse and/or fibrosis. Thoracic CT scan also detected more abnormalities, including consolidation, collapse, nodules, pleural effusion and fibrosis, in both groups. However, the rarity of consolidation and collapse allowed CXR to be as sensitive as thoracic CT scan in detecting cavitary lesions in the PA group, whereas over one-third of the cavitary lesions were undetected in CXR in the CPA group.

### Treatment outcome and prognostic factors

**Figure 2:**
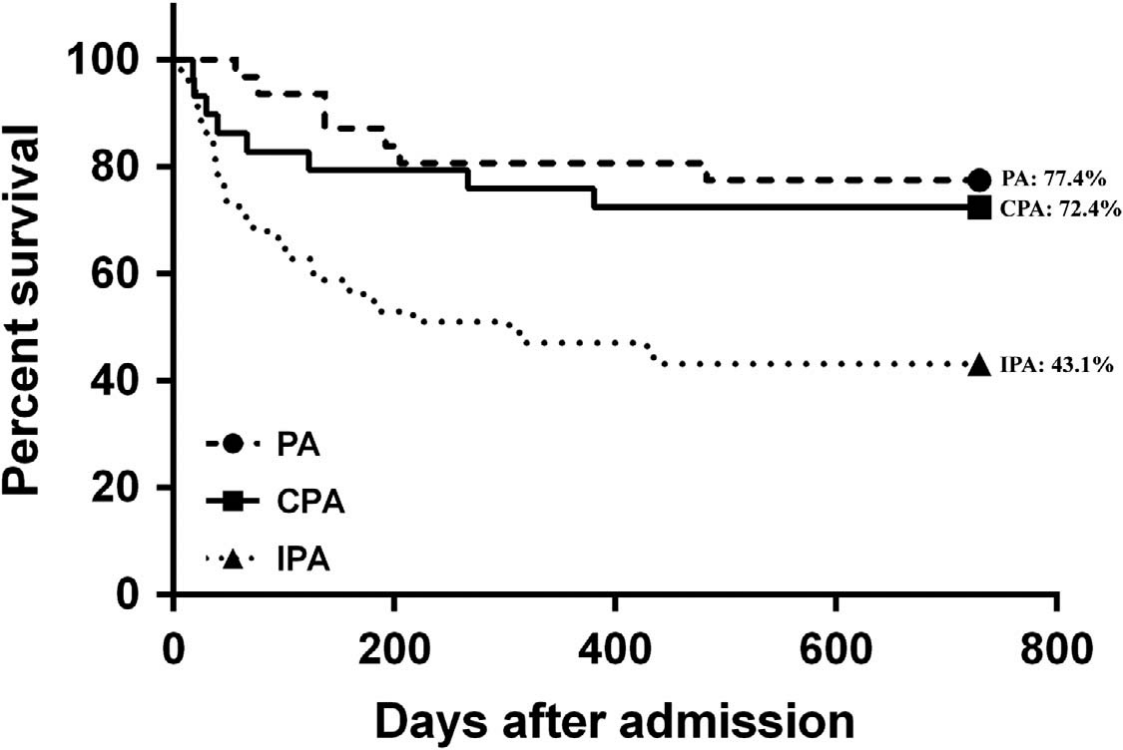
Kaplan–Meier survival curves for CPA, IPA and PA through two years after admission are shown. The values in percentage represent survival rates at 2 years after admission. CPA, chronic pulmonary aspergillosis; IPA, invasive pulmonary aspergillosis; PA, pulmonary aspergilloma.

A significantly higher proportion of the CPA group (100.0%) than the IPA (80.4%) and PA (48.4%) groups received antifungal drugs with anti-aspergillus activity, including itraconazole, voriconazole, posaconazole, caspofungin, micafungin, anidulafungin and/or amphotericin B (Table 2). Nearly 20% of the IPA group did not receive any antifungal treatment before death. The duration of antifungal treatment was longer in the CPA group (median, 176.5 days) than the IPA (median, 42.5 days) and PA (median, 106.0 days) groups, although the difference did not reach statistical significance. None of the PA or CPA patients received surgical resection of an aspergilloma. The duration of hospitalization was significantly shorter in the CPA group (median, 19.0 days) than the IPA group (median, 38.0 days). The survival rates of the CPA group at 30 days, 60 days, 90 days, 180 days, one year, and two years after admission were 89.7%, 86.2%, 82.8%, 79.3%, 75.9% and 72.4%, respectively (Figure 2). The survival rate of the CPA group became significantly higher than that of the IPA group from day 180 (CPA, 79.3% IPA, 54.9% *P*=0.033) onwards until two years after admission (CPA, 72.4% IPA, 43.1% *P*=0.019; Figure 2). The survival rate of the CPA group consistently remained lower than that of the PA group from day 30 (CPA, 89.7% PA, 100.0% *P*=0.107) onwards until 2 years (CPA, 72.4% PA, 77.4% *P*=0.769) after admission, although the difference was not significantly different (Figure 2). The survival rate of CPA (5/8, 62.5%) patients who died from the infection was also intermediate between those who died of PA (1/7, 14.3%) and IPA (22/29, 75.9%). On analysis of the prognostic indicators, subgroup univariate analysis showed that patients who died from CPA within two years after admission were significantly older (69.0±7.1 versus 60.5±17.8 years; *P*=0.019) and had higher total leukocyte (9.3±5.3 versus 6.2±3.7; *P*=0.011) and neutrophil counts (7.7±5.1 versus 4.6±3.9; *P*=0.012) on admission than those who survived. Other laboratory parameters, including lymphocyte and platelet counts, serum levels of sodium, potassium, urea, creatinine, albumin, globulin, bilirubin, alkaline phosphatase, alanine transferase, aspartate transferase and random glucose, and the duration of antifungal treatment and hospitalization, were not significantly different between the death and survival CPA groups. Multivariate analysis showed that a higher total leukocyte count was the only independent prognostic factor (odds ratio=1.230; 95% confidence interval=1.014–1.493; *P*=0.036).

## DISCUSSION

CPA has long been described as an ‘intermediate' or ‘semi-invasive' form of pulmonary aspergillosis.^3, 4, 5^ However, direct comparison of CPA with IPA and PA using results from previous studies that separately assessed these entities was limited by their heterogeneous patient populations, study periods, and laboratory test methods and result interpretations. This 10-year retrospective study was the first to systematically compare the characteristics of hospitalized patients with CPA and those with IPA and PA who were managed in the same institution using a constant set of diagnostic criteria and mycological investigations performed by a single laboratory. We showed that the overall characteristics of CPA more closely resembled those of PA than IPA, and we made novel observations with important implications on the distinction of CPA from IPA and PA, and the assessment of their relative severities based on clinical outcome.

Although a number of novel diagnostic tests have been developed for pulmonary aspergillosis, serum galactomannan antigen and anti-*Aspergillus* antibody remain the most widely used non-invasive tests in most laboratories.^10, 11, 12, 13, 14, 15^ We observed several important phenomena when comparing the performance of serum galactomannan antigen, serum anti-*Aspergillus* antibody, and culture of respiratory tract specimens among the three groups. Firstly, an increasing trend in the seropositive rate of galactomannan antigen from PA (24.1%) to CPA (35.7%) to IPA (54.9%) and an opposite trend for anti-*Aspergillus* antibody (PA, 71.0% CPA, 45.8% IPA, 7.1%) were noted (Table 2). The rates correlated with the degree of host immunosuppression and the invasiveness of the infection in the three groups, and supported the long-standing dogma that CPA represents an intermediate form of pulmonary aspergillosis that lies between the indolent PA and the aggressive IPA. Second, serum galactomannan antigen did not reliably differentiate CPA from IPA and PA, and should not be used to differentiate CPA from IPA or PA. However, serum anti-*Aspergillus* antibody was much more likely to be positive in CPA and PA than in IPA and might be useful in cases where the diagnosis is uncertain. For example, the serum anti-*Aspergillus* antibody test may be useful for overlap syndromes in which more than one form of pulmonary aspergillosis coexists in the same patient and in cases of misdiagnosis of IPA as CPA in patients without classical risk factors, especially critically ill patients in the intensive care unit and patients with chronic obstructive pulmonary disease.^1, 6, 16, 17^ Third, although *A. fumigatus* was consistently the most commonly isolated species in all three groups, >1 species were found in 8.0% of patients with CPA while none with IPA and PA had a mixed infection. This finding might affect the choice of antifungal treatment and highlighted the importance of performing antifungal susceptibility tests, especially in CPA patients who do not respond well to first-line treatment. Finally, we observed a lower seropositive rate of anti-*Aspergillus* antibody in our CPA group than reported in previous studies (45.8% versus ⩾70%).^5, 7^ The apparent difference might be due to various reasons, including the timing of disease as the titer of serum antibody may vary over time or even revert to negative in CPA, the fibrosing form of CPA as IgG serological response may be negative in such patients, and the inclusion of immunocompromised patients who failed to elicit an adequate antibody response.^5^

Similar to previous reports on the radiological appearances of pulmonary aspergillosis, characteristic cavitary lesions were predominantly found in the upper lobes in all three groups in our cohort. However, these lesions were missed in the CXR of more than one-third of the CPA group and one-half of the IPA group, as many had coexisting consolidation, collapse and/or fibrosis. Our findings supported the use of thoracic CT scan in patients with suspected CPA or IPA but not patients with PA with whom CXR and thoracic CT scan were equally sensitive for detecting cavitary lesions. CPA could be differentiated from PA radiologically, as the former frequently appeared as multilobar involvement with various radiological findings not limited to cavitary lesions alone, whereas the latter usually manifested as a unilobar upper lobe cavitary lesion with or without coexisting fibrosis from previous lung damage. CPA might be differentiated from IPA by the presence of pulmonary fibrosis associated with their underlying chronic lung diseases and/or the fibrosing form of CPA.

The clinical outcome of our patients also supported the intermediate severity of CPA (Figure 2). The survival rate of the CPA group remained consistently intermediate between the IPA and PA groups from day 30 up to two years after admission. The survival rate of the CPA group became significantly higher than that of the IPA group from day 180 onwards until two years after admission (*P*<0.05). The prognostic indicators identified in subgroup univariate analysis (older age and higher total leukocyte and neutrophil counts on admission among those who died than those who survived) and multivariate analysis (higher total leukocyte count) suggested that inadequate control of pulmonary inflammation in immunosenescent hosts was essential in determining the outcome of CPA but not in the prognosis of IPA or PA by either univariate or multivariate analysis. Patients with CPA were the most likely among the three groups to receive antifungal treatment (CPA, 100.0% IPA, 80.4% PA, 48.4%), and the median treatment duration was also the longest (CPA, 176.5 days; IPA, 42.5 days; PA, 106.0 days), corroborating the slowly progressive course of CPA, which might improve with long-term antifungal treatment (Table 1).^18^ In contrast, nearly 20% of the patients with the more invasive IPA died before the diagnosis was suspected or shortly after treatment was started, and the less-invasive PA usually did not warrant treatment. In those who survived in the CPA group, a much shorter hospitalization duration for rehabilitation was usually required when compared with those who survived in the IPA group. A significantly higher proportion of CPA patients than IPA and PA patients received itraconazole. This was likely related to the availability and lower cost of the oral preparation of itraconazole. However, amphotericin B and echinocandins were more commonly used among IPA patients, as these drugs required intravenous administration and were more expensive and were reserved for the IPA patients with more severe infections.

Our study had several limitations. First, our study was subject to the intrinsic limitations of retrospective analyses. Nevertheless, this was the first and largest cohort for the systemic comparison of the three major non-allergic forms of pulmonary aspergillosis that provided novel, clinically relevant observations. Second, in contrast to the reported incidence of pulmonary aspergillosis, there were relatively fewer cases of PA, as we only included hospitalized patients to increase the homogeneity of the cohort. As our PA group had characteristics similar to those reported in other studies, we considered them to be representative of PA in general. Third, we did not evaluate the significance of the serum galactomannan antigen index value, as some of the data were not retrievable. Fourth, the sensitivity of the anti-*Aspergillus* antibody assay used in our study may be suboptimal to other newer assays. However, as this 10-year study data were retrospectively collected, we were unable to retest the serum samples with an additional antibody test. Finally, the number of diagnostic tests performed was not uniform in each patient. More opportunities were available for repeated testing in the slowly progressing CPA and PA patients than the rapidly progressing IPA patients. However, we consider our study to be clinically relevant, as the data represent real-life scenarios encountered by clinicians in the diagnosis and management of pulmonary aspergillosis over a long study period.

## Figures and Tables

**Table 1:** Diagnostic criteria of pulmonary aspergilloma, chronic pulmonary aspergillosis and invasive pulmonary aspergillosis in this study

**Category**	**Diagnostic criteria**
*Chronic pulmonary aspergillosis*^a^	All of the following: 1. Chronic pulmonary or systemic symptoms (duration, three months) compatible with chronic pulmonary aspergillosis, including at least one of the following symptoms: weight loss, productive cough or hemoptysis. 2. Cavitary pulmonary lesion with evidence of paracavitary infiltrates, new cavity formation or expansion of cavity size over time. 3. Either positive result of serum *Aspergillus* precipitins test or isolation of *Aspergillus* sp. from pulmonary or pleural cavity. 4. Elevated levels of inflammatory markers (C-reactive protein, plasma viscosity or erythrocyte sedimentation rate). 5. Exclusion of other pulmonary pathogens, by results of appropriate cultures and serological tests, that are associated with similar disease presentation, including mycobacteria and endemic fungi (especially *Coccidioides immitis* and *Histoplasma capsulatum*). 6. No overt immunocompromising conditions (for example, HIV infection, leukemia and chronic granulomatous disease).
	
**Invasive pulmonary aspergillosis**^b^	
Proven	Microscopic analysis (sterile material): histologic, cytologic or direct microscopic examination of a specimen obtained by needle aspiration or biopsy in which hyphae are seen accompanied by evidence of associated tissue damage. Culture (sterile material): recovery of *Aspergillus* sp. by culture of a specimen obtained by a sterile procedure from a normally sterile and clinically or radiologically abnormal site consistent with an infectious disease process, excluding bronchoalveolar lavage fluid.
Probable	**Host factors (any one)**: 1. Recent history of neutropenia (<0.5x10^9^ neutrophils/L for >ten days) temporally related to the onset of fungal disease. 2. Receipt of an allogeneic stem cell transplant. 3. Prolonged use of corticosteroids at a mean minimum dose of 3 mg/kg/day of prednisone equivalent for >three weeks. 4. Treatment with other recognized T-cell immunosuppressants, such as cyclosporine, TNF-α blockers, specific monoclonal antibodies (such as alemtuzumab) or nucleoside analogues during the past 90 days. 5. Inherited severe immunodeficiency (such as chronic granulomatous disease or severe combined immunodeficiency).
	**Clinical criteria**: The presence of one of the following three signs on CT: (1) dense, well-circumscribed lesion(s) with or without a halo sign; (2) air-crescent sign; or cavity.
	**Mycological criteria (any one)**: 1. Direct test (cytology, direct microscopy, or culture): *Aspergillus* sp. in sputum, tracheal aspirate, bronchoalveolar lavage fluid, bronchial brush, indicated by either the presence of fungal elements denoting a mold or recovery by culture of *Aspergillus* sp. 2. Indirect tests (detection of antigen or cell–wall constituents): galactomannan antigen detected in plasma or serum.
Probable without prespecified radiologic findings	Same as for ‘probable' invasive pulmonary aspergillosis except that a positive serum galactomannan antigen result was required, and the abnormal pulmonary infiltrates findings did not fulfill the prespecified radiological criteria.
*Pulmonary aspergilloma*^c^	The presence of a single mobile mass within an existing cavity on chest radiograph or thoracic CT scan, with or without culture of *Aspergillus* sp. from respiratory tract specimens.

Abbreviations: computerized tomography, CT; human immunodeficiency virus, HIV.

a Adopted from Denning *et al.*^5^

b Adopted from De Pauw *et al.*^6^ and Nucci *et al.*^7^ Possible IPA was not included in this study.

c Adopted from Tam *et al.*^8^

**Table 2:** Comparative demographics, mycological investigation results, hospitalization and treatment of patients with chronic pulmonary aspergillosis, invasive pulmonary aspergillosis and pulmonary aspergilloma

**Parameter**	**CPA (*n*=29)**	**IPA (*n*=51)**	**P (CPA versus IPA)**	**PA (*n*=31)**	**P (CPA versus PA)**
Age (years)	64 (7–83)	51 (7–82)	<0.001	66 (40–87)	NS
Sex (male)	25 (86.2)	34 (66.7)	NS	28 (90.3)	NS
*Mycological investigations*					
Serum galactomannan antigen^a^	10 (35.7)	28 (54.9)	NS	7 (24.1)	NS
Serum anti-*Aspergillus* antibody^b^	11 (45.8)	3 (7.1)	<0.001	22 (71.0)	NS
					
**Culture**					
Positive culture from any respiratory tract specimen^c^	25 (86.2)	28 (63.6)	NS	20 (64.5)	NS
					
**Aspergillus species isolated from culture**					
*Aspergillus fumigatus*	17 (68.0)	18 (64.3)	NS	10 (50.0)	NS
*Aspergillus flavus*	2 (8.0)	3 (10.7)	NS	0 (0.0)	NS
*Aspergillus niger*	0 (0.0)	0 (0.0)	NS	3 (15.0)	NS
*Aspergillus terreus*	0 (0.0)	1 (3.6)	NS	1 (5.0)	NS
*Aspergillus* sp.	4 (16.0)	6 (21.4)	NS	6 (30.0)	NS
>1 species	2 (8.0)	0 (0.0)	NS	0 (0.0)	NS
Duration of hospitalization (days)	19.0 (1.0–150.0)	38.0 (1.0–177.0)	0.031	12.0 (1.0–137.0)	NS
					
*Antifungal treatment*					
Use of antifungal drugs	29 (100.0)	41 (80.4)	0.011	15 (48.4)	<0.001
Itraconazole	25	17	<0.001	13	<0.001
Voriconazole	10	19	NS	3	0.028
Posaconazole	0	4	NS	0	NS
Amphotericin B	2	17	0.012	0	NS
Echinocandins	0	15	0.002	1	NS
Duration of antifungal drugs (days)	176.5 (8.0–539.0)	42.5 (1.0–502.0)	NS	106.0 (7.0–411.0)	NS

Abbreviations: bronchoalveolar lavage, BAL; chronic pulmonary aspergillosis, CPA; computerized tomography, CT; chest radiograph, CXR; invasive pulmonary aspergillosis, IPA; left lower lobe, LLL; left upper lobe, LUL; not significant, NS; pulmonary aspergilloma, PA; right lower lobe, RLL; right middle lobe, RML; right upper lobe, RUL.

The data are the number or proportion (%) of patients.

a Serum galactomannan antigen was obtained in 28 (96.6%), 51 (100.0%) and 29 (93.5%) patients with CPA, IPA and PA, respectively.

b Serum anti-*Aspergillus* antibody was obtained in 24 (82.8%), 42 (82.4%) and 31 (100.0%) patients with CPA, IPA and PA, respectively.

c At least one respiratory tract specimen was collected for fungal culture in 29 (100.0%), 44 (86.3%) and 31 (100.0%) patients with CPA, IPA and PA, respectively.

**Table 3:** Comparative radiological investigation results of patients with chronic pulmonary aspergillosis, invasive pulmonary aspergillosis and pulmonary aspergilloma

**Investigation**	**CPA (*n*=29)**	**IPA (*n*=51)**	**P (CPA versus IPA)**	**PA (*n*=31)**	**P (CPA versus PA)**
**Radiological studies**^a^					
Site of CXR abnormality					
RUL	7 (24.1)	5 (9.8)	NS	16 (51.6)	0.036
RML	3 (10.3)	4 (7.8)	NS	0 (0.0)	NS
RLL	1 (3.4)	3 (5.9)	NS	1 (3.2)	NS
LUL	5 (17.2)	5 (9.8)	NS	14 (45.2)	0.027
LLL	0 (0.0)	1 (2.0)	NS	0 (0.0)	NS
Multilobar	13 (44.8)	30 (58.8)	NS	0 (0.0)	<0.001
** CXR features**					
Normal	0 (0.0)	3 (5.9)	NS	0 (0.0)	NS
Cavitary lesion(s)	19 (65.5)	8 (15.7)	<0.001	31 (100.0)	<0.001
Consolidation or collapse	10 (34.5)	31 (60.8)	0.036	2 (6.5)	0.009
Nodule(s)	2 (6.9)	8 (15.7)	NS	5 (16.1)	NS
Pleural effusion	2 (6.9)	9 (17.6)	NS	0 (0)	NS
Fibrosis	13 (44.8)	7 (13.7)	<0.001	19 (61.3)	NS
**Site of thoracic CT scan abnormality**					
RUL	4 (15.4)	3 (9.4)	NS	16 (53.3)	0.005
RML	3 (11.5)	2 (6.3)	NS	0 (0.0)	NS
RLL	1 (3.8)	0 (0.0)	NS	0 (0.0)	NS
LUL	5 (19.2)	3 (9.4)	NS	14 (46.7)	0.047
LLL	0 (0.0)	1 (3.1)	NS	0 (0.0)	NS
Multilobar	13 (50.0)	22 (68.8)	NS	0 (0.0)	<0.001
**Thoracic CT scan features**					
Normal	0 (0.0)	1 (3.1)	NS	0 (0.0)	NS
Cavitary lesion(s), halo or air-crescent sign	26 (100.0)	12 (37.5)	<0.001	30 (100.0)	NS
Consolidation or collapse	12 (46.2)	14 (43.8)	NS	2 (6.7)	0.002
Nodule(s)	6 (23.1)	13 (40.6)	NS	8 (26.7)	NS
Pleural effusion	4 (15.4)	8 (25.0)	NS	2 (6.7)	NS
Fibrosis	14 (53.8)	6 (18.8)	<0.001	23 (76.7)	NS

Abbreviations: bronchoalveolar lavage, BAL; chronic pulmonary aspergillosis, CPA; computerized tomography, CT; chest radiograph, CXR; invasive pulmonary aspergillosis, IPA; left lower lobe, LLL; left upper lobe, LUL; not significant, NS; pulmonary aspergilloma, PA; right lower lobe, RLL; right middle lobe, RML; right upper lobe, RUL.

The data are the number or proportion (%) of patients.

a CXR was performed in all 111 patients. Thoracic CT scan was performed in 26 (89.7%), 30 (96.7%) and 32 (62.7%) patients with CPA, PA and IPA, respectively.

## References

[bib1] Kousha M, Tadi R, Soubani AO. Pulmonary aspergillosis: a clinical review. Eur Respir Rev 2011; 20: 156–174. 21881144 10.1183/09059180.00001011 PMC9584108

[bib2] Yuen KY, Woo PC, Ip MS et al. Stage-specific manifestation of mold infections in bone marrow transplant recipients: risk factors and clinical significance of positive concentrated smears. Clin Infect Dis 1997; 25: 37–42. 9243031 10.1086/514492

[bib3] Gefter WB, Weingrad TR, Epstein DM, Ochs RH, Miller WT. ‘Semi-invasive' pulmonary aspergillosis: a new look at the spectrum of aspergillus infections of the lung. Radiology 1981; 140: 313–321. 7255704 10.1148/radiology.140.2.7255704

[bib4] Binder RE, Faling LJ, Pugatch RD, Mahasaen C, Snider GL. Chronic necrotizing pulmonary aspergillosis: a discrete clinical entity. Medicine 1982; 61: 109–124. 7038373 10.1097/00005792-198203000-00005

[bib5] Denning DW, Riniotis K, Dobrashian R, Sambatakou H. Chronic cavitary and fibrosing pulmonary and pleural aspergillosis: case series, proposed nomenclature change, and review. Clin Infect Dis 2003; 37 (Suppl 3): S265–S280. 12975754 10.1086/376526

[bib6] De Pauw B, Walsh TJ, Donnelly JP et al. Revised definitions of invasive fungal disease from the European Organization for Research and Treatment of Cancer/Invasive Fungal Infections Cooperative Group and the National Institute of Allergy and Infectious Diseases Mycoses Study Group (EORTC/MSG) Consensus Group. Clin Infect Dis 2008; 46: 1813–1821. 18462102 10.1086/588660 PMC2671227

[bib7] Nucci M, Nouer SA, Grazziutti M et al. Probable invasive aspergillosis without prespecified radiologic findings: proposal for inclusion of a new category of aspergillosis and implications for studying novel therapies. Clin Infect Dis 2010; 51: 1273–1280. 21034199 10.1086/657065

[bib8] Tam EW, Chen JH, Lau EC et al. Misidentification of *Aspergillus nomius* and *Aspergillus tamarii* as *Aspergillus flavus*: characterization by internal transcribed spacer, beta-Tubulin, and calmodulin gene sequencing, metabolic fingerprinting, and matrix-assisted laser desorption ionization-time of flight mass spectrometry. J Clin Microbiol 2014; 52: 1153–1160. 24452174 10.1128/JCM.03258-13 PMC3993464

[bib9] Yuen KY, Chan CM, Chan KM et al. Characterization of AFMP1: a novel target for serodiagnosis of aspergillosis. J Clin Microbiol 2001; 39: 3830–3837. 11682494 10.1128/JCM.39.11.3830-3837.2001 PMC88451

[bib10] Woo PC, Leung AS, Lau SK, Chong KT, Yuen KY. Use of recombinant mitogillin for serodiagnosis of *Aspergillus fumigatus*-associated diseases. J Clin Microbiol 2001; 39: 4598–4600. 11797610 10.1128/JCM.39.12.4598-4600.2001 PMC88602

[bib11] Woo PC, Chan CM, Leung AS et al. Detection of cell wall galactomannoprotein Afmp1p in culture supernatants of *Aspergillus fumigatus* and in sera of aspergillosis patients. J Clin Microbiol 2002; 40: 4382–4387. 12409437 10.1128/JCM.40.11.4382-4387.2002 PMC139671

[bib12] Chan CM, Woo PC, Leung AS et al. Detection of antibodies specific to an antigenic cell wall galactomannoprotein for serodiagnosis of *Aspergillus fumigatus* aspergillosis. J Clin Microbiol 2002; 40: 2041–2045. 12037061 10.1128/JCM.40.6.2041-2045.2002 PMC130809

[bib13] Hao W, Pan YX, Ding YQ et al. Well-characterized monoclonal antibodies against cell wall antigen of *Aspergillus* species improve immunoassay specificity and sensitivity. Clin Vaccine Immunol 2008; 15: 194–202. 18032591 10.1128/CVI.00362-07 PMC2238036

[bib14] Lee KC, Tam EW, Lo KC et al. Metabolomics analysis reveals specific novel tetrapeptide and potential anti-inflammatory metabolites in pathogenic *Aspergillus* species. Int J Mol Sci 2015; 16: 13850–13867. 26090713 10.3390/ijms160613850 PMC4490527

[bib15] Meersseman W, Vandecasteele SJ, Wilmer A et al. Invasive aspergillosis in critically ill patients without malignancy. Am J Respir Crit Care Med 2004; 170: 621–625. 15229094 10.1164/rccm.200401-093OC

[bib16] Samarakoon P, Soubani A. Invasive pulmonary aspergillosis in patients with COPD: a report of five cases and systematic review of the literature. Chron Respir Dis 2008; 5: 19–27. 18303098 10.1177/1479972307085637

[bib17] Al-Shair K, Atherton GT, Harris C et al. Long-term antifungal treatment improves health status in patients with chronic pulmonary aspergillosis: a longitudinal analysis. Clin Infect Dis 2013; 57: 828–835. 23788240 10.1093/cid/cit411 PMC3749749

[bib18] Chu CM, Woo PC, Chong KT et al. Association of presence of *Aspergillus* antibodies with hemoptysis in patients with old tuberculosis or bronchiectasis but no radiologically visible mycetoma. J Clin Microbiol 2004; 42: 665–669. 14766834 10.1128/JCM.42.2.665-669.2004 PMC344487

