# Supplementary material for: A 10-year study reveals clinical and laboratory evidence for the ‘semi-invasive' properties of chronic pulmonary aspergillosis
Source: Emerg Microbes Infect. 2016 Apr 20;5(4):e37–. doi: 10.1038/emi.2016.31 (PMC4855073; doi:10.1038/emi.2016.31)
Supplement: Supplementary Table S1 [file emi201631x1.pdf]

**Supplementary Table 1 Underlying Medical Conditions of Patients With Chronic Pulmonary Aspergillosis, Invasive Pulmonary Aspergillosis, and Pulmonary Aspergilloma**

| Variable | CPA (n = 29) | IPA (n = 51) | *P* (CPA vs IPA) | PA (n = 31) | *P* (CPA vs PA) |
|---|---|---|---|---|---|
| **Underlying medical conditions:** | | | | | |
| **Neutropenia** | 0 (0.0) | 21/51 (41.2) | <0.001 | 0 (0.0) | NS |
| **Previous pulmonary tuberculosis** | 20 (69.0) | 5 (9.8) | <0.001 | 25 (80.6) | NS |
| **Active pulmonary tuberculosis** | 0 (0.0) | 1 (2.0) | NS | 0 (0.0) | NS |
| **Chronic lung disease** | 23 (79.3) | 14 (27.5) | <0.001 | 23 (74.2) | NS |
| **Bronchiectasis** | 8 (27.6) | 6 (11.8) | NS | 13 (41.9) | NS |
| **COPD** | 12 (41.4) | 1 (2.0) | <0.001 | 10 (32.3) | NS |
| **Silicosis** | 6 (20.7) | 1 (2.0) | 0.008 | 1 (3.2) | 0.049 |
| **Bronchiolitis obliterans** | 0 (0.0) | 6 (11.8) | NS | 0 (0.0) | NS |
| **Chronic cardiovascular disease** | 6 (20.7) | 11 (21.6) | NS | 5 (16.1) | NS |
| **Chronic renal disease** | 1 (3.4) | 8 (15.7) | NS | 1 (3.2) | NS |
| **Chronic hepatic disease** | 3 (10.3) | 13 (25.5) | NS | 3 (9.7) | NS |
| **Chronic neurological disease** | 1 (3.4) | 0 (0.0) | NS | 1 (3.2) | NS |
| **Diabetes mellitus** | 4 (13.8) | 6 (11.8) | NS | 5 (16.1) | NS |
| **Autoimmune disease**[a] | 2 (6.9) | 6 (11.8) | NS | 0 (0.0) | NS |
| **Corticosteroid or immunosuppressants**[b] | 1 (3.4) | 36 (70.6) | <0.001 | 3 (9.7) | NS |
| **Chemotherapy**[b] | 0 (0.0) | 14 (27.5) | 0.001 | 0 (0.0) | NS |
| **Solid organ malignancy** | 1 (3.4) | 3 (5.9) | NS | 0 (0.0) | NS |
| **Haematological malignancy** | 0 (0.0) | 27 (52.9) | <0.001 | 0 (0.0) | NS |
| **SOT** | 0 (0.0) | 13 (25.5) | 0.003 | 1 (3.2) | NS |
| **HSCT** | 0 (0.0) | 20 (39.2) | <0.001 | 0 (0.0) | NS |
| **HIV infection** | 0 (0.0) | 0 (0.0) | NS | 0 (0.0) | NS |

Data are number or proportion (%) of patients or median (range).
Abbreviations: COPD, chronic obstructive pulmonary disease; CPA, chronic pulmonary aspergillosis; HIV, human immunodeficiency virus; HSCT, haematopoietic stem cell transplantation; IPA, invasive pulmonary aspergillosis; NS, not significant; PA, pulmonary aspergilloma; SOT, solid organ transplantation.
[a]Both patients had ankylosing spondylitis and were not on corticosteroid or immunosuppressants.
[b]Treatment in the preceding 90 days.
